# Change in Nutritional Status and Dysphagia after Resection of Head and Neck Cancer

**DOI:** 10.3390/nu13072438

**Published:** 2021-07-16

**Authors:** Ayumi Sadakane-Sakuramoto, Yoko Hasegawa, Kazuma Sugahara, Nobuhide Horii, Syota Saito, Yuta Nakao, Tomoki Nanto, Takahiro Ono, Kazuhisa Domen, Hiromitsu Kishimoto

**Affiliations:** 1 Department of Dentistry and Oral Surgery, Hyogo College of Medicine, 1-1 Mukogawa-cho, Nishinomiya 663-8501, Hyogo, Japan; ayu.cherry.ayu@gmail.com (A.S.-S.); kazumaro0526@gmail.com (K.S.); no-horii@hyo-med.ac.jp (N.H.); kisihiro@hyo-med.ac.jp (H.K.); 2 Division of Comprehensive Prosthodontics, Faculty of Dentistry and Graduate School of Medical and Dental Sciences, Niigata University, Niigata 951-8514, Japan; ono@dent.niigata-u.ac.jp; 3 Department of Physical Medicine and Rehabilitation, Hyogo College of Medicine College Hospital, Nishinomiya 663-8501, Hyogo, Japan; hardler_popo@yahoo.co.jp (S.S.); ynakao33@gmail.com (Y.N.); tomokinanto@gmail.com (T.N.); 4 Okamura Clinic, Nishinomiya 662-0912, Hyogo, Japan; 5 Department of Rehabilitation Medicine, Hyogo College of Medicine, Nishinomiya 663-8501, Hyogo, Japan; domen@hyo-med.ac.jp

**Keywords:** nutritional status, head and neck cancer, prognostic nutritional index, dysphagia

## Abstract

Nutritional status is well-known to influence patient recovery after resection of head and neck cancer (HNC). The influence of preoperative nutritional status on dysphagia was assessed in patients who underwent surgical resection of HNC along with the assessment of nutritional status during the acute and subacute phases. Eighty-six patients underwent surgical resection and dysphagia assessments (repetitive saliva-swallowing test, water-swallowing test, and functional oral intake scale) and had their tongue pressure assessed five times (before surgery, after 1–2 weeks, and 1, 2, and 3 months after surgery). The nutritional status was assessed according to the body mass index, total protein, and albumin. The prognostic nutritional index was calculated from preoperative data, and the subjects were classified into three groups: Low-risk, Attention and High-risk groups. After surgery, the nutritional status index values were low, and the High-risk group showed significantly lower values in comparison to the other two groups. The water-swallowing test and functional oral intake scale findings were worse than they had been preoperatively until 2 months after surgery, and a significant correlation was noted between the postoperative nutritional status and the presence of dysphagia. The results indicated that the preoperative nutritional status of HNC patients influenced their ability to ingest/swallow, which in turn influenced their nutritional status after HNC resection.

## 1. Introduction

After surgical resection for patients with head and neck cancer (HNC), malnutrition is likely to occur due to oral intake restrictions, stress, and increased protein catabolism due to invasive surgery. As a result, issues such as muscle weakness, delayed wound healing, and postoperative infections are likely to occur [1]. Patients with HNC may be preoperatively malnourished due to tumor-induced protein consumption in addition to a loss of appetite [2,3], with many already suffering from nutritional disorders at the time of admission. Furthermore, patients with HNC may already have severe malnutrition prior to surgery, so surgery without careful consideration in malnourished cases can sometimes lead to serious complications [4].

As performing surgery on malnourished patients without careful consideration can sometimes lead to serious complications, in order to improve the prognosis of HNC surgery, it is important to have a good grasp of their nutritional status beforehand, as well as before and after HNC resection, and manage nutrition after surgery; in other words, an assessment of nutrition should be conducted before and after surgery [5]. Continuous nutritional assessment from preoperative to postoperative status is expected to reduce the risk of postoperative malnutrition, accelerate wound healing after HNC resection, and lead to early recovery.

It is well known that there is a correlation between pre-HNC resection nutritional status/immune status and post-HNC resection complication rate/postoperative recovery [5]. To date, we have conducted continuous assessments of the dysphagia status in patients who underwent HNC resection and reported changes in their dysphagia status [6,7]. We have reported that among HNC resection patients, those with suprahyoid muscle group resection [7], advanced cancer [6], and those who have combined radiation therapy, cervical dissection, and reconstruction are more likely to have dysphagia. As the ingesting/swallowing function of HNC patients is greatly reduced immediately after surgery, the risk of aspiration is high immediately after surgery [6,7]. Furthermore, regarding dysphagia after HNC surgery, it is known that oral function will be restored, thereby leading to weight gain and improvement of nutritional status, by performing appropriate intervention after HNC resection, such as performing treatments for palatal augmentation prosthesis and jaw prosthodontic [8], in addition to performing feeding/swallowing rehabilitation [8]. On the other hand, there are no reports on the temporal changes in nutritional status before and after HNC resection, so the evidence is insufficient.

The purpose of this prospective study was to evaluate the changes over time in the dysphagia and nutritional status before and after surgery in patients after resection of head and neck cancer and to elucidate the association between the nutritional status and dysphagia status from the acute phase to the subacute phase.

## 2. Materials and Methods

This study was approved by the institutional ethics committee at Hyogo College of Medicine (H26-1713). Patients who participated in this study gave their informed consent in writing after receiving a sufficient description of the research before registering for inclusion.

### 2.1. Subjects

Patients who underwent surgical resection of HNC at the Department of Oral and Maxillofacial Surgery and Otolaryngology, Hyogo Medical College, between August 2013 and December 2014. After the patient had given consent for surgical resection of the HNC, we checked the medical records and conducted additional consultations as required to determine eligibility for the study. The inclusion criteria were as follows: (1) providing informed consent to participate in the study and (2) being available for regular follow-up visits after surgery. The exclusion criteria were as follows: (1) severe dementia and/or a decline in the cognitive function with a preoperative revised Hasegawa’s dementia scale ≤20 points [9]; (2) tumor lesion of unknown primary; (3) withdrawal from the study due to relocation/transfer to another hospital or other reasons; (4) a history of radiotherapy and/or chemotherapy prior to surgery; (5) a history of complications including dysphagia (i.e., stroke or neuromuscular disease). We had explained this study to patients, and those who gave their written informed consent to participate in this prospective study were included.

The primary tumor lesion and tumor progression were evaluated using the Union for International Cancer Control tumor/node/metastasis (TNM) and stage classification [10]. If the attending physician diagnosed the need for oral appliances (surgical obturator, removal denture, palatal augmentation prosthesis, and/or palatal lift prosthesis), the patient was allowed to wear these appliances (adjusted by the dentist) in a manner similar to daily living, and the dysphagia screening test described below was administered.

### 2.2. Assessment Schedule and Evaluators

We prospectively collected all data for this study after obtaining informed consent. The dysphagia status was evaluated five times: before surgery (range: day −12 to −1), 1–2 weeks after surgery (day +2 to +14), 1 month after surgery (day +21 to +41), 2 months after surgery (day +46 to +79), and 3 months after surgery (day +83 to +115). In all items, items that could not be evaluated were treated as missing values. All potential evaluators (dentists, speech therapists, and dental hygienists) listened to a trained speech therapist (assessment experience of 12 years and certified by the Japanese Society of Dysphagia Rehabilitation) deliver a lecture on all dysphagia assessment methods, and then the evaluators underwent assessment training twice (2 h/day, total of 4 h) before starting this study. To avoid measurement bias, evaluators who performed assessments were blinded to the objective of this study. This study’s evaluations were conducted by dentists, speech therapists, and dental hygienists, each of whom was involved in the treatment of the target patients.

### 2.3. Nutritional Status

We evaluated the nutritional status according to the body mass index (BMI), total protein (TP), and serum albumin (Alb) [11,12,13]. The data of ±3 days at the time of nutritional function evaluation were used for the analysis. BMI was classified into three groups: underweight, normal, and obese, based on the WHO criteria for obesity. Upon confirming the values of the TP, Alb (g/dL), and total lymphocytes count (TLC (/mm^3^), based on the blood sampling data, the prognostic nutritional index (PNI) was given in order to assess the nutritional prognosis [14]. Blood was collected within ±2 days before and after the dysphagia test.

PNI was calculated from the albumin level and the number of lymphocytes, using the PNI calculation formula proposed by Onodera et al. [14].

PNI = (10 × Alb) + (0.005 × TLC)

### 2.4. Dysphagia Screening Tests

**Figure 1:**
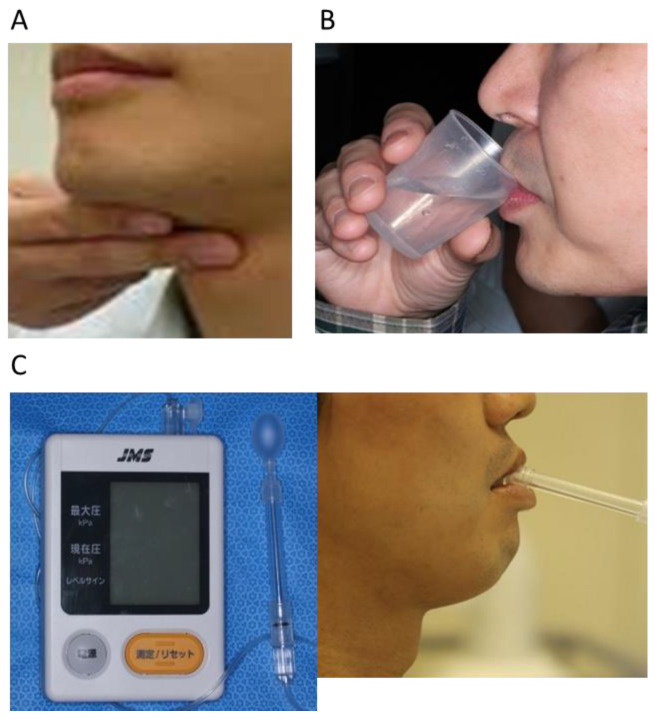
Assessments of dysphagia. (**A**) Repetitive swallow saliva test (RSST). Palpation of the laryngeal elevation with the index and middle fingers. (**B**) Water-swallowing test (WST). The patient was asked to drink 30 mL water as usual. (**C**) Tongue pressure: the patient was asked to raise their tongue and compress the balloon onto the palate with maximal voluntary effort for approximately 7 s.

The dysphagia screening tests were performed according to the evaluation criteria of the Japanese Society of Dysphagia Rehabilitation. We performed the repetitive saliva-swallowing test (RSST) [6,15,16] and water-swallowing test (WST) [6,17] (Figure 1).

The RSST is used to study the ability of the patient to spontaneously and repeatedly swallow, which is highly associated with aspiration [6,15,16]. The RSST is a simple test and relatively safe to carry out. The patient was placed in a resting position, and the inside of the mouth was moistened with cold water. The patient was instructed to repetitively swallow air and to count up the number of swallows completed. The number of swallows was counted visually and by palpation of the laryngeal elevation with the index and middle fingers.

For the WST, the patient was seated in a chair and handed a cup containing 30 mL of room temperature water. The patient was instructed to drink the water as usual. The time taken to empty the cup was measured, and the drinking profile and episodes were monitored and evaluated as: (1) the patient could drink all of the water in one gulp without coughing; (2) the patient could drink all of the water in two or more gulps without coughing; (3) the patient could drink all of the water in one gulp, but with some coughing; (4) the patient could drink all of the water in two or more gulps, with some coughing; or (5) the patient often coughed and had difficulty drinking all of the water [18].

### 2.5. Functional Oral Intake Scale (FOIS)

Ref. [19] to classify the severity of dysphagia, all patients underwent an assessment of their oral condition, with classification conducted according to the FOIS [20,21] (Table 1).

### 2.6. Tongue Pressure Measurement

Tongue pressure measurements were conducted with the JMS tongue pressure measurement system and were assessed according to the method reported by Tsuga et al. [22]. We previously showed that tongue pressure measurement was a safe, useful, and objective tool for assessing dysphagia immediately postoperatively in patients with head and neck cancer [7,21]. The examiner instructed the patient to sit while relaxed and placed the balloon on the anterior part of the palate. To remove the effect of the power of the mandible closing via the anterior teeth or residual ridge, the end of a plastic cylinder crossed the teeth/ridge arch. The patient was then asked to raise the tongue and compress the balloon onto the palate as strongly as possible with maximal voluntary effort for approximately 7 s. For patients with a reconstruction of the tongue, the tongue pressure was measured by placing a balloon between the center of the remaining tongue and the palate. The measurements were taken three times, and the mean value was adopted as the tongue pressure.

### 2.7. Data Collection and Statistical Analyses

Based on the preoperative PNI value, the subjects were determined to be in the Low-risk group when the PNI was ≥50 and the Attention group when 40 ≤ PNI < 50, with High risks of excision/anastomosis (hereinafter, the High-risk group) when PNI < 40 [5]. With regard to the preoperative nutritional status and dysphagia conditions, the Kruskal–Wallis test was used to examine the differences of PNI between groups. After that, the Mann–Whitney U test was used to compare between the groups. (*p*-value was corrected by Bonferroni’s method.)

The time courses of the nutritional status and dysphagia condition were evaluated using a linear mixed-effects model with the main effects of time and PNI group (High risk/Attention/Low risk) and their interaction effect as fixed effects. The time effect was regarded as a categorical variable (preoperative, 1–2 weeks after surgery, 1 month after surgery, 2 months after surgery, and 3 months after surgery). When significant differences in main effects were found, multiple comparisons (post hoc test) were performed using the Mann–Whitney-U test. *p* values of Mann–Whitney-U test were corrected using Bonferroni’s method. Spearman’s rank-correlation coefficient was used to analyze the relationships between the nutritional status and the dysphagia condition before and after surgery.

We performed all statistical analyses using the SPSS statistics software program, version 22.0 (IBM, Tokyo, Japan). A value of *p* ≤ 5% was judged to be statistically significant.

## 3. Results

Table 2 shows an overview of the subjects.

Data are shown as the number of patients (percentage of all subjects). Cancer treatment indicates the number of patients who underwent surgery. For chemotherapy and radiotherapy, the number of patients who received adjuvant treatment postoperatively is indicated.

The origin of primary HNC varied, with tongue cancers being the most common in 26 cases (30.2%), followed by gingival cancers in 22 cases (25.6%), and hypopharyngeal cancers in 15 cases (17.4%). The degree of cancer progression in each patient was highest in stage IV (40.7%), while stage III or higher accounted for 50% of the subjects. More than 30% of patients underwent reconstruction or tracheotomy at the time of HNC resection, while 38.4% of the subjects underwent cervical dissection.

Table 3 shows the preoperative nutritional status and dysphagia conditions of the subject patients.

Based on the nutritional status of preoperative patients, 53 patients were diagnosed and assigned to the Low-risk group with 50 <= PNI or more, 19 patients to the Attention group with 40 <= PNI <= 50, requiring attention in perioperative management, and 14 patients to the High-risk group with PNI < 40. The Attention group and the High-risk group together accounted for 38.4% of the total, and we could not say that the preoperative nutritional status of the subjects in this study was good. Significant differences were observed for TP and Alb among the three groups of PNI, indicating that the High-risk group had poor nutritional status before surgery, especially compared with the other two groups. There was no significant difference in BMI and FOIS. No difference was observed between the groups in terms of other dysphagia conditions, indicating that there was no difference between the groups in terms of preoperative swallowing function.

**Figure 2:**
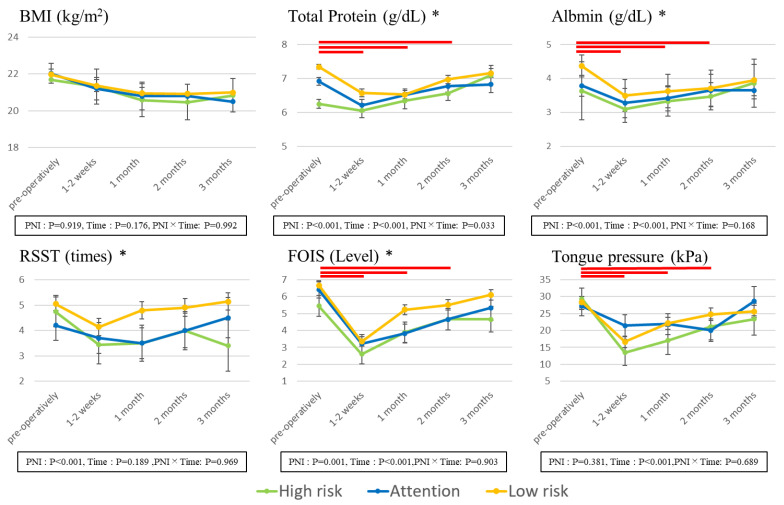
Changes in the nutritional status and condition of dysphagia. BMI, body mass index (kg/m^2^); albumin, serum albumin; RSST, repetitive swallow saliva test, FOIS; functional oral intake scale. Data are shown as the mean ± S.E. The lower box *p*-value shows the results of the linear mixed-effects model with the main effects of time (preoperatively, and at 1–2 weeks, 1 month, 2 months, and 3 months postoperatively) and PNI group (High risk, Attention, Low risk) and their interaction effect as fixed effects. When significant differences in main effects were found, multiple comparisons (post hoc test) were performed using the Mann-Whitney-U test. *p* values were corrected using Bonferroni’s method. The bar indicates that there is a significant difference in comparison to the preoperative value. *: Significant difference between the contraindication and Low-risk groups. There were no groups that differed significantly from the Attention group in any items.

Figure 2 illustrates the results of a comparison of nutritional status and dysphagia conditions, with preoperative and postoperative changes over time and between the three groups of PNI.

The BMI and total protein values were significantly lower at 1–2 weeks/1 month/2 months after surgery than before surgery. These values tended to decrease after surgery compared to before surgery, indicating that the recovery to preoperative body weight was not easy after HNC surgery. Furthermore, the High-risk group had significantly lower values of TP and Alb than the Low-risk group, indicating that nutritional status is difficult to recover even after surgery.

With regard to the dysphagia status, FOIS and tongue pressure indicated significantly lower values 1–2 weeks/1 month/2 months after surgery compared to before surgery, and the swallowing function declined for the duration of 2 months after surgery, indicating that it was difficult to take nutrition orally. With regard to RSST and FOIS, the values of the High-risk group were significantly lower than those of the Low-risk group, suggesting that it is difficult for subjects with nutritional status problems to recover their swallowing function even after surgery. As a result of comparing between three groups of PNI, there were significant differences between three groups of TP and Alb. With regard to FOIS, the values of the High-risk group were significantly lower than those of the Low-risk group.

Table 4 shows the correlation between preoperative and postoperative nutritional status (PNI, BMI, TP, Alb) and dysphagia condition (RSST, FOIS, WST, tongue pressure).

There was a weak correlation between FOIS and nutritional status (PNI, BMI, Alb) before surgery; however, after surgery, there was a significant association between the swallowing function, especially RSST/FOIS/WST, and the nutritional status other than BMI (PNI, TP, Alb) in many cases, indicating that the swallowing state and nutritional state may be more strongly associated with each other after surgery than before surgery. Tongue pressure was weakly associated with nutritional status compared to the other three swallowing function assessments.

## 4. Discussion

### 4.1. Assessment of the Dysphagia

The significant correlation between the dysphagia status and nutritional status on several variables was found before surgery, suggesting that the risk of undernutrition is high even before surgery. Correlations were found between WST and FOIS in the screening test and nutritional endpoints excluding BMI, with a correlation between tongue pressure and BMI, similar to before surgery. It was speculated that there was a stronger correlation between the dysphagia status and nutritional status after surgery than before surgery.

With regard to the assessment of the dysphagia condition, while instrumental testing, such as videofluoroscopy [20] and a fiberoptic endoscopic evaluation of swallowing [23] is the gold standard, there were many patients for whom these methods could not be performed due to the degree of postoperative rest. Furthermore, HNC patients with large surgical margins cannot undergo VFSS or FEES from the acute to subacute phase, making it impossible to evaluate temporal changes in postoperative dysphagia continuously. The dysphagia assessment test used in the present study can easily be performed at the bedside as a screening tool for dysphagia in almost all patients; for this reason, we adopted the dysphagia screening test in the present study.

With regard to the assessment of the dysphagia status, including tongue pressure, similar to our previous reports [6,7], it was indicated that the risk of aspiration was very high immediately after surgery because it decreased significantly immediately after surgery [6]. In addition, the tongue pressure was reduced immediately after surgery, with the level of reduction dependent on the primary tumor site [7].

The FOIS is widely used as an index of the nutritional intake and has the advantage of being able to be used to make continuous assessments, from tube feeding to oral food intake. In addition, the FOIS has already been reported for its reproducibility and validity [24]. Several studies have used the FOIS for HNC patients [25,26], so it was also adopted in the present study.

Regarding the presence of intraoral appliances, 31 (36%) patients wore removable dentures (maxilla/mandible/both) preoperatively, 9 (10%) patients wore new palatal prosthetics (splint immediately after surgery or obturator with denture) after surgery, and 4 (4.5%) patients wore a palatal augmentation prosthesis fabricated after surgery. Patients who wore removable dentures were evaluated with their appliance in place from the preoperative to the postoperative state, and postoperative denture repairs were performed as needed. Therefore, the effects of intraoral appliances on the assessment of the dysphagia status were considered to be small, as no new removable dentures were fabricated. However, it is possible that patients who donned new prosthetics (e.g., obturator prosthesis, palatal augmentation prosthesis) fabricated after surgery had an advantage in some assessments [27] There have been reports of significant changes in tongue pressure but no significant change in RSST in patients with a palatal augmentation prosthesis [28]. Removable dentures and/or prostheses were worn at the time of the assessments because the main purpose of this study was to evaluate the ingestion and swallowing function and nutrition in the acute and subacute phases and the prosthesis was necessary to close the nasopharynx and smooth the oral nutrition intake.

### 4.2. Nutrition Assessment

Regular reassessment of nutritional status is required during postoperative management in order to perform nutritional management based on nutritional assessment. It is desirable to correct the trajectory of the nutritional management method (re-planning) by emphasizing the changes over time in each nutritional assessment while performing nutritional management [19]. Although there are many indicators for performing nutritional assessment, it may be difficult to make a comprehensive judgment because some indicators show abnormalities, while some parameters remain within the normal range.

As a result of this study, it was found that although FOIS is an assessment of the nutrition intake method and dietary pattern of patients, it has a strong correlation with the assessment by PNI, with FOIS found to be lower in the group of patients who had lower PNI than before surgery, resulting in a major obstacle to eating. It was believed that the Low-risk group needed more consideration regarding dietary patterns than before surgery due to the surgery, and although the High-risk group exhibited a tendency to improve compared to before surgery, some dietary modification was performed after discharge, with the PNI value indicating that the condition required attention in management even after discharge.

### 4.3. Nutritional Indicators Used in This Study

The BMI is also an indirect method of estimating body fat mass [12,13]. Blood protein concentration TP well reflects the dynamics of visceral protein and is important as an index for assessing nutritional status [11,12], while Alb is a protein synthesized in the liver, accounting for approximately 60% of blood protein. Albumin has a long half-life of approximately 21 days, and because there are many extravascular pools, fluctuations in blood concentration are small; therefore, while it cannot be said to indicate short-term nutritional dynamics, it can be used as an index for long-term nutritional management [12]. In addition, serum albumin level is a routine examination after surgical resection of HNC; it had been reported to be a useful indicator of the impact of surgery on physical function in patients with gastrointestinal cancer [29].I It reflects not only nutritional status but also postoperative biological changes such as protein catabolism associated with surgical invasion. For these reasons, serum albumin level was used as a nutritional index in this study.

PNI was proposed by Buzby et al. in 1980 [2]. It is a prognosis estimation formula derived from a preoperative nutrition index that predicts the occurrence of postoperative complications via a single formula [2]. It is an index to evaluate systemic nutritional status more comprehensively, using variables that combine various nutritional assessments. Subsequently, PNI was proposed by Onodera et al. as well [14]. The PNI proposed by Onodera et al. is used in many facilities because it is determined using only two factors, blood albumin level and peripheral lymphocyte count, in addition to being simple and relatively reliable [11,12]. The PNI estimation formula proposed by Onodera et al. was adopted this time as appropriate for assessment because the subjects were also Japanese, who were the index for Onodera et al.

Furthermore, because it is being used not only for advanced malignant tumors but also for malignant diseases in general, the change over time in the prognostic nutrition index was assessed in this study using the calculation for PNI proposed by Onodera et al. The nutritional assessment by Onodera et al. uses the albumin level and lymphocyte count, with the advantage of being able to be easily calculated from a normal blood test. In addition, PNI is known to help predict postoperative complications and prognosis [5]. Protein catabolism increases during recovery from the inflammatory state of the human body, including surgical invasion, leading to malnutrition and eventually delayed wound healing. Therefore, it is necessary to order protein-enriched infusion during postoperative management or dealing with critically ill patients such as those in the ICU. Similarly, in patients with head and neck cancer, protein catabolism increases due not only to surgery but also the restriction of oral intake because of the primary disease, stress, or invasive surgery, potentially resulting in malnutrition, muscle weakness, delayed wound healing, and postoperative infection [5]. Understanding and maintaining preoperative PNI is useful in predicting postoperative complications for improvement even in patients with head and neck cancer and is the key to postoperative nutritional management.

### 4.4. Study Limitations

This study involved patients with head and neck cancer without classifying them. Namely, the patients in the study were very diverse and heterogeneous in terms of primary tumor lesion/operative procedure/application range of resection. It has been reported that the degree of dysphagia in head and neck cancer varies depending on the site of origin and the degree of progression, which is a topic for further study. Furthermore, although the data from the acute phase to the convalescent phase after surgery was used, the data for patients who were discharged or transferred early were not included because the course had not been completed.

The surgical procedures varied among patients, and some patients developed mucositis due to chemotherapy and/or radiation therapy after surgical resection [25,26]. Data analyses with defects were performed using a linear mixing model, but there were some cases in which a postoperative evaluation was impossible due to dysphagia symptoms. In the acute phase, the tactile bone is less likely to touch due to postoperative swelling of the cervical region, so conducting the RSST can become difficult. In patients who have undergone tongue resection, the tongue pressure may not be evaluable due to pain. For example, the use of a probe-type system to measure the tongue pressure was difficult since this measurement was affected by the number of residual teeth and the amount of tongue remaining, and such measurement was technically impossible in patients who underwent subtotal lingual resection. Several patients complained of strong pain during tongue pressure measurement. As such, the type/location of surgery (e.g., surgery for tongue cancer) may hamper or prevent tongue pressure measurement [25].

### 4.5. Clinical Implications

The result of this study indicated that not only is the nutritional status poor after surgery, but the dysphagia status is also not restored when the preoperative PNI is in a bad state as High risk. In other words, if the preoperative PNI is at High risk, surgical resection should be reconsidered, and if PNI requires performing HNC surgery on patients with High risk, it can be inferred that sufficient preoperative rehabilitation planning is necessary for postoperative nutritional maintenance and recovery. Depending on the surgical site of HNC, it is possible to restore the oral function by performing prosthetic treatment and rehabilitation such as jaw dentures [8]; a restored oral function makes it possible for patients to eat food, thereby leading to weight gain and improved nutritional status. Therefore, it may be one plan to strategically restore oral function after surgery.

## 5. Conclusions

It was indicated that the preoperative nutritional status of patients with head and neck cancer affects the swallowing function and nutritional status after HNC resection.

In particular, dysphagia and malnutrition occur easily, up to 2 months after surgery, indicating the importance of preoperative nutritional management and rehabilitation. As it is not easy for patients after discharge to recover their weight before surgery, continuous evaluation of their nutritional status and dietary guidance is considered necessary. If no weight gain is observed after surgery, it is expected that the intake will decrease due to decreased oral function, indicating that postoperative treatment planning and rehabilitation planning before surgery are important.

## Figures and Tables

**Table 1:** Functional oral intake scale.

**LEVEL7**	Total oral feeding with no restrictions
**LEVEL6**	Oral feeding, multiple consistencies, no special preparation, specific food limitations
**LEVEL5**	Oral feeding, multiple consistencies, requiring special preparation or compensations
**LEVEL4**	Total oral feeding of a single consistency
**LEVEL3**	Tube dependent with consistent oral intake of food or liquid
**LEVEL2**	Tube dependent, minimal attempts of food or liquid
**LEVEL1**	NPO

The FOIS assesses the patient’s nutritional intake method and dietary form [19]. The FOIS scores ranged from Level 1 (nothing by mouth) to Level 7 (total oral feeding with no restrictions).

**Table 2:** Patient characteristics.

All Patients (N = 86)		*n* (%)
General	Gender	
	Male	57 (66.3)
	Female	29 (33.7)
	Age ± S.D.	67.2±13.5
Cancer details		
*Primary site*	Maxillary sinus	1 (1)
	Gingiva	22 (25.6)
	Buccal mucosa	6 (7.0)
	Tongue	26 (30.2)
	Floor of oral cavity	5 (5.8)
	Oropharynx	9 (10.5)
	Hypopharynx	15 (17.4)
	Larynx	2 (2.3)
*Stage*	I	15 (20.9)
	II	26 (30.2)
	III	10 (11.6)
	IV	35 (40.7)
*Cancer treatment*	Chemotherapy	12 (14.0)
	Radiotherapy	10 (11.6)
	Reconstruction	29 (33.7)
	Tracheotomy	33 (38.4)
	Cervical dissection	
	Bilateral	9 (10.5)
	Unilateral	24 (27.9)

**Table 3:** Preoperative patient conditions.

	High Risk (14 Patients)	Attention (19 Patients)	Low Risk (53 Patients)	Difference between Groups
Nutritional status				
BMI (kg/m^2^)	21.7 ± 3.5	22 ± 3.0	22 ± 2.6	N.S.
Total Protein (g/dL)	5.6 ± 1.1	6.9 ± 0.4	7.2 ± 0.8	a,b,c
Serum Albumin (g/dL)	3.2 ± 0.5	3.8 ± 0.3	4.5 ± 0.7	a,b,c
Dysphagia conditions				
RSST (times)	4.2 ± 2	4.2 ± 1.9	5.0 ± 2.0	N.S.
WST (score)	1.8 ± 1.3	1.8 ± 1.2	1.6 ± 1.0	N.S.
FOIS (level)	5.4 ± 2.6	6.4 ± 1.5	6.7 ± 1.0	N.S.
Tongue pressure (kPa)	29.3 ± 9.8	27.2 ± 9.4	28.4 ± 11.1	N.S.

Data are shown as the mean ± S.D. or median (min–max). Patients were classified into three groups (High-risk, Attention, and Low-risk groups) according to the preoperative prognostic nutritional index. Differences between groups: Kruskal–Wallis test. N.S.: No significant difference. a/b/c: When there was a significant difference in the Kruskal–Wallis test, there was a significant difference in the Mann–Whitney U test (*p*-value was adjusted by Bonferroni’s method), a: High-risk group and Low-risk group, b: Attention group and Low-risk group, c: High-risk group and Attention group. BMI, body mass index; RSST, repetitive saliva-swallowing test; WST, water-swallowing test; FOIS, functional oral intake scale.

**Table 4:** Correlation between nutritional status and dysphagia.

	PNI	BMI	TP	Alb
Pre-operation				
RSST (times)	0.18	−0.04	0.15	0.15
*p*-value	0.13	0.7	0.22	0.22
FOIS (level 1–7)	0.24 *	0.37 **	0.13	0.33 **
*p*-value	0.04	0.003	0.28	0.01
WST (score 1–5)	−0.28 *	−0.04	−0.02	−0.14
*p*-value	0.02	0.77	0.86	0.25
Tongue pressure (kPa)	0.07	0.40 **	−0.15	0.13
*p*-value	0.54	*p* < 0.001	0.21	0.26
Post-operation				
RSST (times)	0.23 **	0.09	0.18	0.49 **
*p*-value	0.003	0.18	0.0	*p* < 0.001
FOIS (level 1–7)	0.39 **	0.05	0.36 **	0.59 **
*p*-value	*p* < 0.001	0.45	*p* < 0.001	*p* < 0.001
WST (score 1–5)	−0.31 **	−0.13	−0.36 **	−0.50 **
*p*-value	*p* < 0.001	0.05	*p* < 0.001	*p* < 0.001
Tongue pressure (kPa)	−0.06	0.25 **	−0.05	0.30 **
*p*-value	0.44	*p* < 0.001	0.53	*p* < 0.001

PNI: prognostic nutritional index, BMI: body mass index, TP: total protein, Alb: serum albumin, RSST, repetitive saliva-swallowing test; WST, water-swallowing test; FOIS, functional oral intake scale. Data are shown as Spearman’s rank-correlation coefficients and *p*-value. * *p* < 0.05, ** *p* < 0.01.

## Data Availability

The data of this study are available on request from the corresponding author, Y.H. The data are not publicly available due to restrictions, as they contain information that could compromise the privacy of the research participants.

## References

[1] Van Der Schuer V.B.-D., Van Leeuwen P.A., Kuik D.J., Klop W.M., Sauerwein H.P., Snow G.B., Quak J.J. (1999). The impact of nutritional status on the prognoses of patients with advanced head and neck cancer. Cancer.

[2] Buzby G.P., Mullen J.L., Matthews D.C., Hobbs C.L., Rosato E.F. (1980). Prognostic nutritional index in gastrointestinal surgery. Am. J. Surg..

[3] Usami M., Fukuda A., Miyoshi M., Yamamoto I., Takahashi M. (2013). Nutritional Therapy for Cancer Patients. Shikoku Acta Med..

[4] Sato M. (1982). The clinical study of nutritional evaluation with gastric cancer patients. Jpn. Cent. Rev. Med..

[5] Furudoi S., Yoshii T., Hayashi T., Miyai D., Yoshikawa T., Komori T. (2000). Correlation between Prognostic Nutritional Index (PNI) and Postoperative Infections in Oral Cancer. J. Jpn. Stomatol. Soc..

[6] Horii N., Hasegawa Y., Sakuramoto-Sadakane A., Saito S., Nanto T., Nakao Y., Domen K., Ono T., Kishimoto H. (2021). Validity of a dysphagia screening test following resection for head and neck cancer. Ir. J. Med Sci..

[7] Hasegawa Y., Sugahara K., Fukuoka T., Saito S., Sakuramoto A., Horii N., Sano S., Hasegawa K., Nakao Y., Nanto T. (2017). Change in tongue pressure in patients with head and neck cancer after surgical resection. Odontology.

[8] Kreeft A.M., Van Der Molen L., Hilgers F.J., Balm A.J. (2009). Speech and swallowing after surgical treatment of advanced oral and oropharyngeal carcinoma: A systematic review of the literature. Eur. Arch. Oto-Rhino-Laryngol..

[9] Kato S., Shimogaki M., Onodera A., Ueda H., Oikawa K., Ikeda K., Kosaka A., Imai M., Hasegawa K. (1991). Creating a revised Hasegawa Dementia Scale (HDS-R). Jpn. J. Geriatr. Psychiatry.

[10] Sobin L.H., Mary K. (2010). Gospodarowicz, Christian Wittekind. TNM Classification of Malignant Tumours.

[11] Sungurtekin H., Sungurtekin U., Balci C., Zencir M., Erdem E. (2004). The Influence of Nutritional Status on Complications after Major Intraabdominal Surgery. J. Am. Coll. Nutr..

[12] Higashiguchi T. (2005). The Basics and Practice of Nutritional Therapy. NST Perfect Guide.

[13] Prado C.M.M., Heymsfield S.B. (2014). Lean Tissue Imaging: A new era for nutritional assessment and intervention. J. Parenter. Enter. Nutr..

[14] Kanda M., Fujii T., Kodera Y., Nagai S., Takeda S., Nakao A. (2011). Nutritional predictors of postoperative outcome in pancreatic cancer. J. Br. Surg..

[15] Baba M., Saitoh E., Okada S. (2008). Dysphagia Rehabilitation in Japan. Phys. Med. Rehabil. Clin. N. Am..

[16] Sakayori T., Maki Y., Hirata S., Okada M., Ishii T. (2013). Evaluation of a Japanese “Prevention of Long-term Care” project for the improvement in oral function in the high-risk elderly. Geriatr. Gerontol. Int..

[17] Osawa A., Maeshima S., Tanahashi N. (2013). Water-Swallowing Test: Screening for Aspiration in Stroke Patients. Cerebrovasc. Dis..

[18] Tohara H., Saitoh E., Mays K.A., Kuhlemeier K., Palmer J.B. (2003). Three Tests for Predicting Aspiration without Videofluorography. Dysphagia.

[19] Crary M.A., Mann G.D.C., Groher M.E. (2005). Initial Psychometric Assessment of a Functional Oral Intake Scale for Dysphagia in Stroke Patients. Arch. Phys. Med. Rehabil..

[20] Arrese L.C., Schieve H.J., Graham J.M., Stephens J.A., Carrau R.L., Plowman E.K. (2019). Relationship between oral intake, patient perceived swallowing impairment, and objective videofluoroscopic measures of swallowing in patients with head and neck cancer. Head Neck.

[21] Hamahata A., Beppu T., Shirakura S., Hatanaka A., Yamaki T., Saitou T., Sakurai H. (2014). Tongue pressure in patients with tongue cancer resection and reconstruction. Auris Nasus Larynx.

[22] Tsuga K., Yoshikawa M., Oue H., Okazaki Y., Tsuchioka H., Maruyama M., Yoshida M., Akagawa Y. (2012). Maximal voluntary tongue pressure is decreased in Japanese frail elderly persons. Gerodontology.

[23] Hiss S.G., Postma G.N. (2003). Fiberoptic Endoscopic Evaluation of Swallowing. Laryngoscope.

[24] Kunieda K., Ohno T., Fujishima I., Hojo K., Morita T. (2013). Reliability and Validity of a Tool to Measure the Severity of Dysphagia: The Food Intake LEVEL Scale. J. Pain Symptom Manag..

[25] Tashimo Y., Ihara Y., Yuasa K., Nozue S., Saito Y., Katsuta H., Shimane T., Takahashi K. (2019). Acute Stage Longitudinal Change of Quality of Life from Pre- to 3 Months after Surgical Treatment in Head and Neck Cancer Patients. Asian Pac. J. Cancer Prev..

[26] Van den Steen L., Van Gestel D., Vanderveken O., Vanderwegen J., Lazarus C., Daisne J.-F., Van Laer C., Specenier P., Van Rompaey D., Mariën S. (2019). Evolution of self-perceived swallowing function, tongue strength and swallow-related quality of life during radiotherapy in head and neck cancer patients. Head Neck.

[27] Marunick M., Tselios N. (2004). The efficacy of palatal augmentation prostheses for speech and swallowing in patients undergoing glossectomy: A review of the literature. J. Prosthet. Dent..

[28] Okayama H., Tamura F., Kikutani T., Kayanaka H., Katagiri H., Nishiwaki K. (2008). Effects of a palatal augmentation prosthesis on lingual function in postoperative patients with oral cancer: Coronal section analysis by ultrasonography. Odontology.

[29] Hara T., Igawa T., Sano M., Shinomiya M., Nakano T., Matsuzawa M., Ishii T., Matsumoto K., Yoshida C., Sakurai A. (2014). Serum Albumin is a Marker of Physical Function in Perioperative Gastrointestinal Cancer Patients. Rigakuryoho Kagaku.

